# Orthodontic Treatment and Maxillary Anterior Segmental Distraction Osteogenesis of a Subject with Williams–Beuren Syndrome and Isolated Cleft Palate: A Long-Term Follow-Up from the Age of 5 to 24 Years

**DOI:** 10.1155/2017/7019045

**Published:** 2017-07-04

**Authors:** Tetsutaro Yamaguchi, Tatsuo Shirota, Mohamed Adel, Masahiro Takahashi, Shugo Haga, Ryo Nagahama, Misato Nakashima, Mayu Furuhata, Takaaki Kamatani, Koutaro Maki

**Affiliations:** ^1^Department of Orthodontics, School of Dentistry, Showa University, 2-1-1 Kitasenzoku, Ohta-ku, Tokyo 145-8515, Japan; ^2^Department of Oral and Maxillofacial Surgery, School of Dentistry, Showa University, 2-1-1 Kitasenzoku, Ohta-ku, Tokyo 145-8515, Japan

## Abstract

Williams–Beuren syndrome (WBS) is a rare multisystem disorder caused by a hemizygous deletion of the elastin gene on chromosome 7q11.23. WBS patients have characteristic skeletal features and dental anomalies accompanied by mental retardation, a friendly outgoing personality, and mild to moderate intellectual disability or learning problems. In this case report, we present the combined orthodontic and surgical treatment of a WBS patient with an isolated cleft palate through a long-term follow-up from the age of 5 to 24 years. During the period of active treatment, comprehensive orthodontic treatment combined with maxillary anterior segmental distraction osteogenesis and prosthetic treatment using dental implants were effective in dramatically improving the patient's malocclusion. The patient's mental abilities and the cooperation shown by the patient and her family were crucial for the success of this complex and long-term treatment course.

## 1. Introduction

Williams–Beuren syndrome (WBS) is a rare multisystem disorder caused by a hemizygous deletion of the elastin gene on chromosome 7q11.23 [1]. In 1961, Williams et al. were the first to draw attention to the syndrome, with a cardiology report describing a condition with a distinctive range of symptoms including supravalvular aortic stenosis, mental retardation, and dysmorphic facial features. Several years later, Beuren et al. described the syndrome independently and suggested that this condition has dental anomalies as a consistent component [2, 3]. WBS is estimated to affect one in 20,000 individuals with equal prevalence in males and females [4].

WBS is characterized by mental and growth retardation, a friendly outgoing personality, and mild to moderate intellectual disability or learning problems. Individuals with WBS have distinctive facial features and heart and blood vessel (cardiovascular) problems [4, 5]. The typical dysmorphic facial features of WBS are considered to be diagnostic of patients with WBS and include soft tissue and skeletal components. Patients with WBS usually have full prominent cheeks, a full nasal tip, a wide mouth, a long philtrum, and full lips. These facial features are summarized in the term “elfin face” [1, 5]. Four skeletal features contribute to this unique facial appearance: a short cranial base; a steep angle of the mandibular plane; unusual proportions of the upper to lower anterior facial height and the posterior to anterior facial height; and a deficient chin, although the mandible is not classified as retrognathic [6]. Dental abnormalities of WBS include microdontia, hypodontia, abnormal incisor morphology, tongue thrusting, excessive interdental spacing, a higher prevalence of Class II and Class III occlusion, open and deep bites, and anterior crossbite [7]. Cleft palate has never been considered part of the WBS syndrome, but the condition has been little investigated [8].

This case report presents the long-term orthodontic follow-up and treatment of a female patient with WBS and an isolated cleft palate from the age of 5 to 24 years.

## 2. Case Presentation

A female patient with a chronological age of 5 years and 1 month at the time of the first examination presented at the Department of Orthodontics, Showa University Dental Hospital, with a chief complaint of a masticatory disorder. The medical history revealed that the patient had hypercalcemia, a ventricular septum defect, and mental disability and had undergone cleft palate closure surgery at the age of 1 year and 6 months.

### 2.1. Clinical Examination

**Figure 1:**
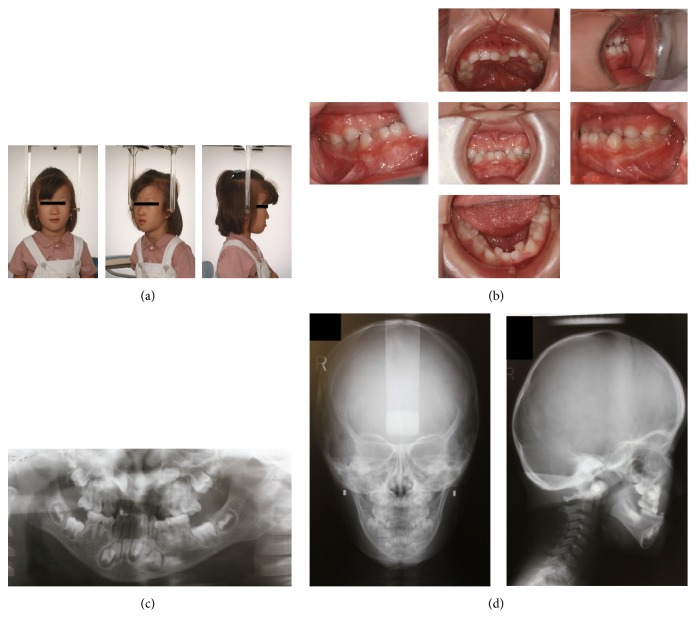
Pretreatment photographs (5 years and 1 month). (a) Extraoral photographs. (b) Intraoral photographs. (c) Orthopantomograms. (d) Posteroanterior and lateral cephalograms.

On extraoral clinical examination, the patient was found to have an “elfin face” appearance, with a small nose, long philtrum, prominent lips, and zygomatic flattening, all of which are distinctive to WBS (Figure 1(a)). Intraorally, the patient had an anterior crossbite and a deep overbite (Figure 1(b)). Pretreatment radiographs included lateral and posteroanterior (PA) cephalograms and orthopantomograms (OPGs) (Figures 1(c) and 1(d)). The OPG showed that the bilateral maxillary second premolars and the bilateral mandibular first and second premolars were congenitally absent (Figure 1(c)).

### 2.2. Facial Protraction Stage

**Figure 2:**
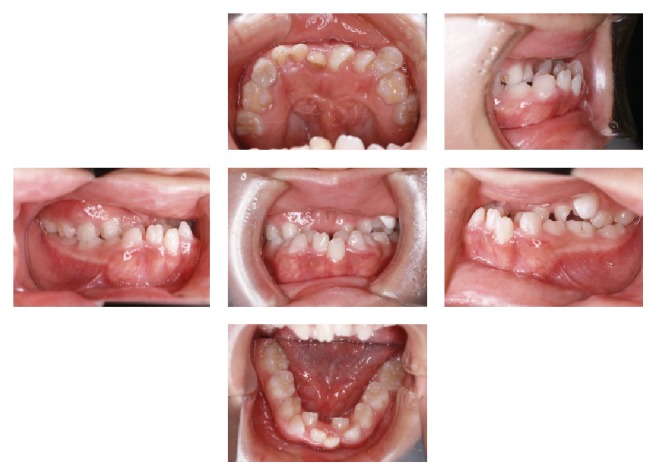
Intraoral photographs before the facemask treatment (9 years and 4 months).

**Figure 3:**
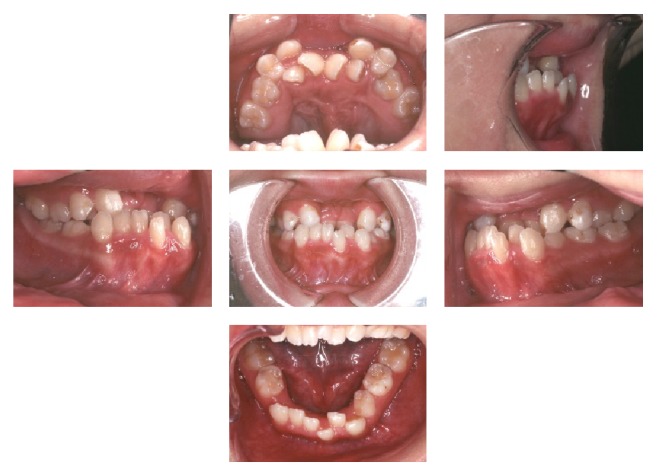
Intraoral photographs during the observation period and after the facemask treatment (13 years and 1 month).

At the age of 9 years and 4 months the patient was reexamined clinically and radiographically. Intraoral examination showed that the overjet was −12.0 mm and the overbite was +3.0 mm (Figure 2). Cephalometric examination (Table 1) revealed a skeletal Class III relationship (ANB −5.0°). To reduce the severe anterior crossbite, facemask therapy was initiated at the age of 11 years and continued for 1 year and 9 months. Thereafter, the patient was regularly followed up three times each year until the next stage of treatment (Figure 3).

### 2.3. Treatment Plan

After the monthly meeting with the Department of Oral and Maxillofacial Surgery, Showa University Dental Hospital, maxillary anterior segmental distraction osteogenesis (MASDO) with predistraction and postdistraction orthodontics was planned. The objective of the treatment was to improve the facial esthetics of the patient and to establish an occlusion that allowed for normal function.

### 2.4. Predistraction Orthodontics Stage

**Figure 4:**
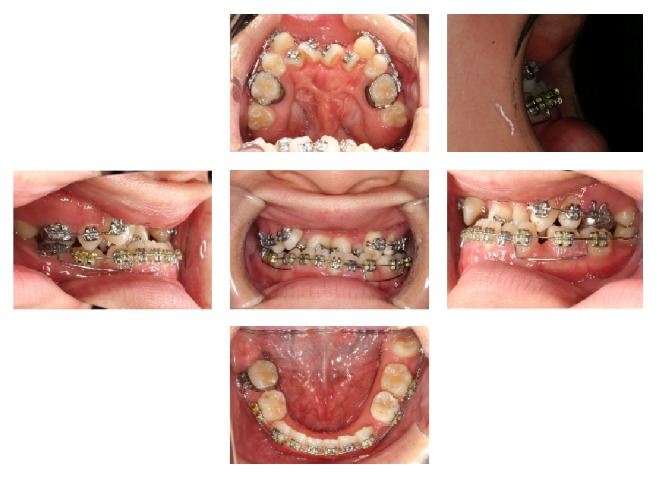
Intraoral photographs during predistraction orthodontic treatment.

At the age of 16 years and 7 months, intraoral examination revealed a Class III molar relationship, an overjet of −7 mm, and severe anterior crowding. Cephalometric examination revealed a skeletal Class III relationship (ANB −3.9°), with excessive lingual inclination of the lower incisors (Table 1). Predistraction orthodontic treatment was commenced in both arches, using 0.018-inch slot preadjusted edgewise appliances. This treatment was continued for 1 year and 11 months and was carried out in a manner similar to the preparation for conventional orthognathic surgery for an Angle Class III occlusal relationship. This included coordination of the arches, decompensation of the anterior dentition, and leveling and alignment. Because of the severe anterior crowding in the upper arch, the orthodontic treatment was confined to the posterior segment to prepare the dentition for the use of the intraoral appliance required for the distraction. After leveling of the lower arch, a utility arch made of 0.016 × 0.016-inch stainless steel wire was fitted to initiate intrusion of the lower incisors and minimize the overbite (Figure 4).

### 2.5. Distraction Osteogenesis Stage

**Figure 5:**
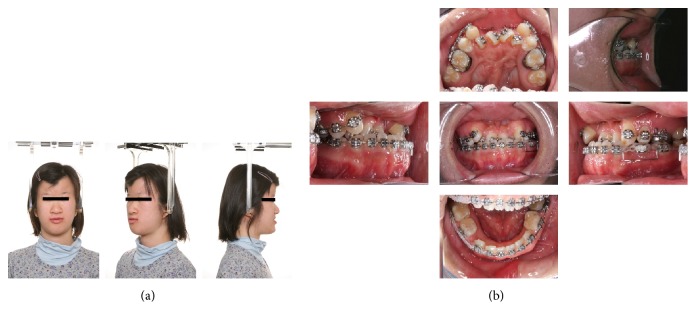
Facial (a) and oral (b) photographs before maxillary anterior segmental distraction osteogenesis (18 years and 6 months).

**Figure 6:**
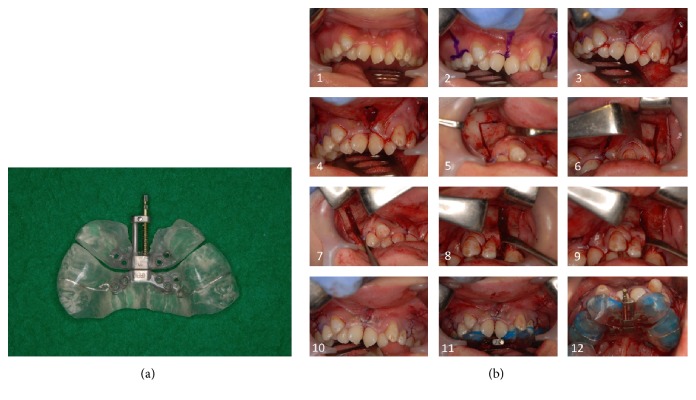
Maxillary anterior segmental distraction osteogenesis (MASDO). (a) Distractor. (b) The numbers indicate the order of the procedures.

After completion of the predistraction orthodontics, impressions were taken of the upper and lower arches (Figure 5). A maxillary biteplate (distractor) was constructed to cover the occlusal surfaces of the posterior teeth and extend anteriorly to the lateral incisors. The distractor was 2.0 mm thick in the posterior region. A horizontal cut was made in the acrylic between the first premolar and first molar areas. An expansion screw oriented anteroposteriorly with an opening capacity of 15.0 mm was inserted into the plate (Figure 6(a)). At the age of 18 years and 6 months, the MASDO was carried out. The surgical approach for distraction was similar to a Le Fort 1 osteotomy. A circumvestibular flap was raised, a complete osteotomy was performed, and the maxilla was then downfractured. The flap was then closed primarily and the distractor was cemented to the teeth (Figure 6(b)). After a latency period of 7 days, the distraction was initiated by turning the screw twice a day (0.5 mm/d) until adequate forward movement was obtained. The consolidation period took 6 months, after which the distractor was removed.

### 2.6. Postdistraction Orthodontics Stage

**Figure 7:**
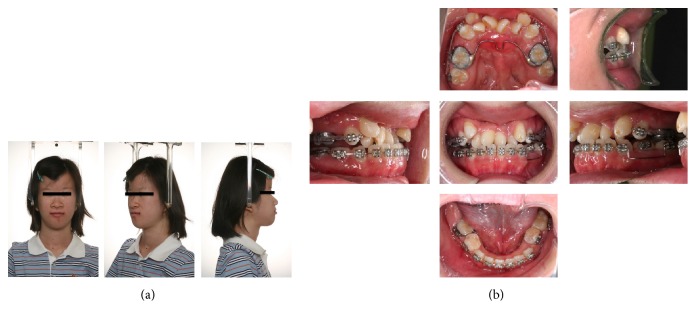
Facial (a) and oral (b) photographs after the consolidation period (19 years).

**Figure 8:**
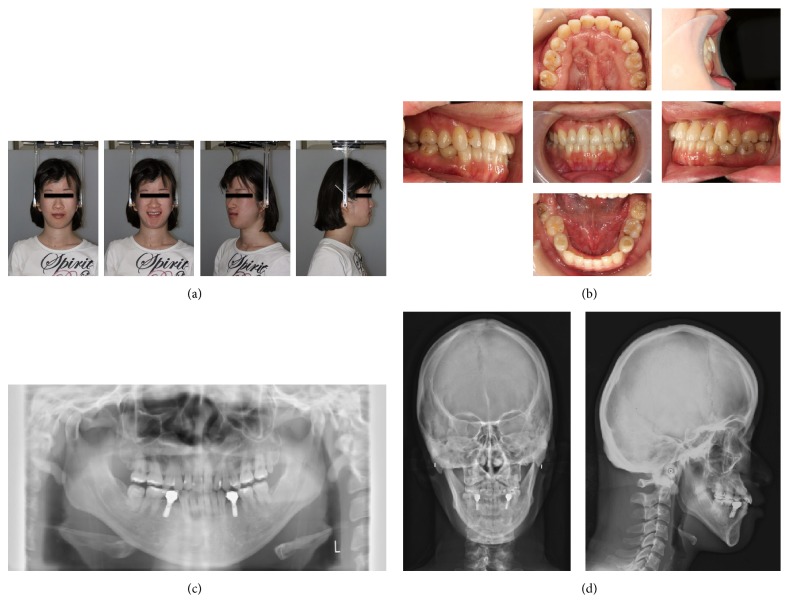
Extraoral and intraoral photographs at 2 years and 8 months after debonding (24 years and 9 months). (a) Extraoral photographs. (b) Intraoral photographs. (c) Orthopantomograms. (d) Posteroanterior and lateral cephalograms.

After the consolidation period, the distracted anterior segment was stable, and neither the teeth nor the soft tissue showed any signs of complications. The distractor was removed and a Nance appliance was placed so that postdistraction orthodontic treatment could resume (Figure 7). The goal of the treatment was to achieve an ideal occlusion concerning the canine class, molar class, overbite, overjet, and coincident dental midline. A sectional 0.016 × 0.016-inch stainless steel wire was placed in the posterior segment to allow the retraction of the premolar into the space created by the distraction process. After completion of the premolar retraction, complete leveling of the maxillary teeth, closure of the residual spaces, and coordination of the arches were carried out. Before the end of the treatment, the lower wisdom teeth were extracted. Thereafter, the fixed appliance was removed at the age of 22 years and 1 month, and Hawley type retainers were fitted in the upper and lower arches. Prosthetic treatment of the area corresponding to the bilateral mandibular premolars was completed using dental implants, and observation of the patient's condition has continued (Figure 8).

## 3. Discussion

Combined surgical and orthodontic treatment typically enhances facial esthetics and improves functional occlusion. Such changes have a positive effect on the patient's quality of life. Orthodontic treatment planning for patients with WBS requires special consideration because the patient has specific skeletal deformities and dental malformations, as well as distinctive mental and psychological behavior. In the present report, the patient had six permanent teeth congenitally absent and an isolated cleft palate, which is seldom a feature of WBS [8]. MASDO significantly improved the anterior crossbite and aided in relieving the crowding. The prognosis for the prosthetic treatment using dental implants was favorable.

The literature is inconsistent about the caries rate in children with WBS. However, the dental treatment of patients with WBS depends basically on their cooperation. Sedation may be helpful in the younger age group to reduce anxiety and uncooperative behavior during treatment for minimal caries. However, treatment under general anesthesia is usually complicated by the medically compromised conditions accompanying WBS [9]. Few case reports of orthodontic treatment in WBS can be found in the literature, which could be attributed to the varying degree of cooperation provided by the patient and family in response to orthodontic treatment. Vieira et al. [10] reported a case of a patient with WBS who received orthodontic treatment with satisfying results. The patient received orthopedic expansion of the maxilla, in which a modified facial mask was used to protract the maxillary complex associated with clockwise rotation of the maxilla. They also concluded that there are alternative ways to manage syndromic patients effectively, depending on the degree of cooperation from the patient and family, as well as the direction and magnitude of facial growth. Fortunately, our patient's mental disability was not severe, and enough family cooperation was provided. Therefore, a favorable treatment result could be achieved, although long-term and complicated treatment procedures were carried out.

In addition to oral and facial characteristics, WBS patients may present with microdontia, generalized diastemas, anodontia, caries, enamel hypoplasia, dental malocclusion, atypical deglutition, and counterclockwise rotation of the maxilla accompanied by a retruded mandible [10]. The present case had a Class III malocclusion associated with a cleft palate and dental agenesis. Although the treatment time extended from the age of 5 to 24 years, almost no carious lesions were detected during the treatment. Additionally, there were no signs of enamel hypoplasia or atypical deglutition.

**Figure 9:**
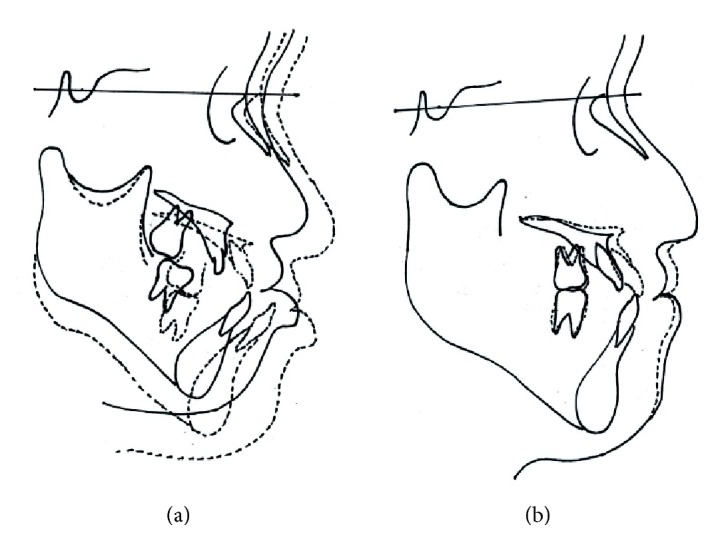
(a) Superimposed cephalometric tracing at pretreatment (solid line) and posttreatment (dotted line) of facemask therapy. (b) Superimposed cephalometric tracing at pretreatment (solid line) and posttreatment (dotted line) of MASDO.

The cephalometric features of WBS often include an anterior inclination of the maxilla, a high mandibular plane angle, and a deficient bony chin [11]. In our case, the cephalometric analysis revealed severe counterclockwise rotation of the maxilla accompanied by a retruded mandible. Parfsch et al. [12] studied the growth data of 244 German children with WBS. They found that girls with WBS were divided into two groups: an early puberty group and a late puberty group. Unfortunately, we did not record body height data for this patient, so we have no information about her growth rate, but she could have had a premature and abbreviated pubertal growth spurt. Superimposed cephalometric tracings at pretreatment and posttreatment of the facemask therapy suggest that the patient experienced late maxillary growth (Figure 9, Table 1). Because of the presence of various oral and maxillofacial features in patients with WBS, most cases require an interdisciplinary treatment approach. Early determination of treatment objectives and the timing of interdisciplinary strategies are important factors for adequate management [11].

The MASDO procedure has been used as an alternative treatment option for patients with severe midfacial deficiency [13–15]. Furthermore, MASDO is effective in increasing alveolar arch length and correcting anterior crossbite in patients with a cleft lip and palate [13, 14, 16]. An advantage of MASDO is that there is no risk of deterioration of velopharyngeal function; this is an occasional complication of a conventional maxillary advancement surgical procedure. Severe anterior crowding in the upper arch is usually relieved by therapeutic extraction of teeth. Tooth extraction was not appropriate in our case because multiple teeth were congenitally missing. MASDO successfully provided the required maxillary alveolar arch length, allowing the maxillary anterior teeth to be properly aligned.

## 4. Conclusion

Complex and individualized treatment planning is required for WBS patients because they exhibit specific skeletal deformities and dental malformations. Special consideration must be given to the degree of cooperation shown by the patient and the family, as well as the patient's mental and psychological behavior, as these are critical factors during treatment.

## Figures and Tables

**Table 1:** Cephalometric analysis at various stages of treatment.

	Initial	Before face mask treatment	After face mask treatment	Before MASDO	Retention	2 years and 8 months after debonding
	September 1995	July 1999	April 2003	October 2008	August 2012	April 2015
Angular (°)						
SNA	82.7	81.5	84.6	84.9	89.3	87.7
SNB	85.2	86.4	86.5	88.9	87.8	86.4
ANB	−2.6	−5	−1.2	−3.9	1.5	1.3
Mp-FH	41.8	37.1	37.5	34.1	34.1	34.6
Gonial angle	138.5	136.4	129.7	126.8	128.3	129.7
U1-FH	92.6	99.9	117.6	114.6	123	126.8
L1-Mp	72	64.6	87.4	76.2	74.6	76.1
Facial angle	82.2	87.6	87.8	92.6	92.1	90.4
Convexity	−4	−7.5	−4.3	−6.8	3.5	3.7
A-B plane	3.5	7.3	1.8	5.1	−1.5	−1
*y*-axis	68.3	63.7	65.7	62.8	63.1	64.5
Linear (mm)						
N-S	51.6	56	58.8	58.4	58.4	58.6
N-Me	90.7	100.6	115.7	120.2	121.7	123.5
N-ANS	37.7	42.1	49.9	51.3	48.5	50.4
ANS-Me	53.4	58.7	66.3	69.4	75.2	74.7
S′-Ptm′	23.9	26.2	30.4	26.8	28.6	26.3
A′-Ptm′	31	35.4	34.8	37.5	32.4	35.8
Gn-Cd	85.9	94.4	107.7	114.7	114.3	114.5
Pog′-Go	55.7	61.2	72.5	75.6	73.9	72.9
Cd-Go	38.3	41.2	48.4	52.8	53.5	54.8

MASDO: maxillary anterior segmental distraction osteogenesis.
